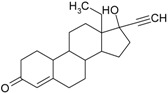# Emerging Estrogenic Pollutants in the Aquatic Environment and Breast Cancer

**DOI:** 10.3390/genes8090229

**Published:** 2017-09-15

**Authors:** Sylvain Lecomte, Denis Habauzit, Thierry D. Charlier, Farzad Pakdel

**Affiliations:** Research Institute for Health, Environmental and Occupational Health (IRSET), U1085 Inserm, TREC Team, University of Rennes, 35000 Rennes, France; sylvain.lecomte@univ-rennes1.fr (S.L.); denis.habauzit@univ-rennes1.fr (D.H.); thierry.charlier@univ-rennes1.fr (T.D.C.)

**Keywords:** endocrine disrupting chemical, estrogen receptor, estrogens, zebrafish, breast cancer

## Abstract

The number and amount of man-made chemicals present in the aquatic environment has increased considerably over the past 50 years. Among these contaminants, endocrine-disrupting chemicals (EDCs) represent a significant proportion. This family of compounds interferes with normal hormonal processes through multiple molecular pathways. They represent a potential risk for human and wildlife as they are suspected to be involved in the development of diseases including, but not limited to, reprotoxicity, metabolic disorders, and cancers. More precisely, several studies have suggested that the increase of breast cancers in industrialized countries is linked to exposure to EDCs, particularly estrogen-like compounds. Estrogen receptors alpha (ERα) and beta (ERβ) are the two main transducers of estrogen action and therefore important targets for these estrogen-like endocrine disrupters. More than 70% of human breast cancers are ERα-positive and estrogen-dependent, and their development and growth are not only influenced by endogenous estrogens but also likely by environmental estrogen-like endocrine disrupters. It is, therefore, of major importance to characterize the potential estrogenic activity from contaminated surface water and identify the molecules responsible for the hormonal effects. This information will help us understand how environmental contaminants can potentially impact the development of breast cancer and allow us to fix a maximal limit to the concentration of estrogen-like compounds that should be found in the environment. The aim of this review is to provide an overview of emerging estrogen-like compounds in the environment, sum up studies demonstrating their direct or indirect interactions with ERs, and link their presence to the development of breast cancer. Finally, we emphasize the use of in vitro and in vivo methods based on the zebrafish model to identify and characterize environmental estrogens.

## 1. Introduction

Breast cancer is the most commonly diagnosed cancer in women and is the second highest cause of death by cancer in women in Western countries. About 50,000 cases of breast cancer are recorded each year in France and the Breast Cancer Society estimates over 250,000 new cases in the USA for 2017. In general, the incidence of hormone-dependent cancers has increased over the past 30 years in industrialized countries [1,2]. Although age is one of the primary risk factors for the development of breast cancer, this increase is not only due to the aging of the population as it is observed in all age groups. In addition, more than 80% of these cancers are not associated with genetic mutations. Environmental factors, including lifestyle, exposure to contaminants, and diet, are likely the main etiological factors [3,4]. Hormonal over-exposure, especially to the estrogen 17β estradiol (E2), is the major risk factor: early menarche and late menopause as well as hormone replacement therapies and elevated levels of circulating E2 in pre-menopausal women are associated with an increased risk of breast cancer [5,6]. Exposure to exogenous hormonal-mimetics such as endocrine-disrupting chemicals (EDCs) is also likely to represent a major risk factor.

**Figure 1:**
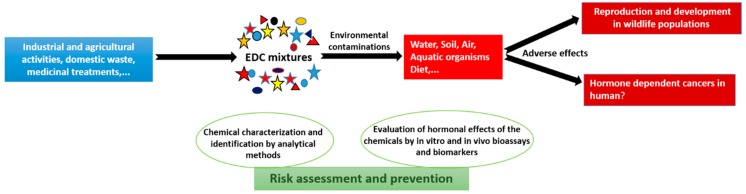
Risk assessment and chemical exposure prevention. Chemical mixtures such as endocrine-disrupting chemicals (EDCs), released from different sources, can be found in the environment and food. They should be monitored through analytical and screening approaches. New detection methods are also needed to quantify risks to animal and human health prior to exposure to the chemical mixtures.

These EDC compounds that interfere with the signaling pathways of endogenous hormones are released in the environment. For example, a large number of pesticides, cosmetics, and phthalates are currently found in surface water and numerous persistent organic pollutants (POPs) are not only found in water, but can accumulate in fat-rich tissues and are found in large concentrations in fish, shrimp, and shellfish. Some of these compounds, called xenoestrogens, mimic endogenous estrogens by binding to and activating estrogen receptors (ERs) and, among other effects, promote mammary cell proliferation, increasing the risk of initiating cell transformation and the development of cancer [7,8]. Furthermore, some heavy metals such as cadmium (Cd) may also act as EDCs and trigger hormone-dependent breast tumorigenesis [9]. Therefore, risk assessment of potential estrogen-mimetics acting on human health should include both the identification and characterization of each active substances present in the environment, but we should also investigate the interactions, or cocktails, of several compounds on patho-physiological processes. As illustrated in Figure 1, chemical mixtures including EDCs are released from industrial and agricultural activities, as well as from domestic wastes and medicinal treatments, and can be found in the environment and food. These molecules, alone and in combination, should be monitored by analytical and biological approaches, as well as alternative screening methods, to quantify risks to animal and human health prior to exposure (Figure 1). Unfortunately, the association between EDC exposures and hormone-dependent cancers or human disorders is not clearly established. One reason for the lack of a definitive association between estrogen-like compounds and health is the little or no epidemiological research to link exposure to EDCs with the early onset of certain diseases, such as the development of ovary and breast cancers. There are also very few studies that examine a possible link between fetal exposure to EDCs and cancers that occur later in adults. Even with the European Union regulation REACH (Registration, Evaluation and Authorisation of Chemicals), the effects of many environmental chemicals have not yet been tested, and satisfactory test methods need to be validated for many diseases and hormonal cancers. Finally, it should be noted that humans are exposed not only to one, but to a mixture of EDCs [10].

## 2. Emerging Environmental Estrogen-Like Compounds

Over the last 50 years the use of chemical pollutants and their release in the environment has increased considerably, triggering major concerns about their impact on wildlife and humans. These environmental concerns have been acknowledged by many institutions, however industrial development, agrochemicals and human chemical consumption produce an increasing amount of chemical pollutants into the environment, especially in surface water. With the general low biodegradability (Table 1) of a number of these molecules, there is an accumulation of the chemicals in the environment. Therefore, the presence of these molecules constitutes a risk for human and wildlife. Among the hundreds of these man-made molecules, those with endocrine-disrupting activities draw peculiar attention due to their potentially disrupting activities at very low doses on human and fauna, and because of their potential link with disorders and diseases.

Besides current natural hormones including 17 β-estradiol (E2), estrone (E1), and estriol (E3), and pharmaceuticals like the contraceptives ethinylestradiol (EE2) and levonorgestrel that are found in concentrations up to ng/L in surface water, several families of chemicals are raising new concerns because of the increased scientific evidence of endocrine-disrupting activity, the lack of a legal definition as such, and their significant presence in the environment. These compounds originate from industrial and household applications and include alkylphenols, used mainly in surfactants and detergents, insecticides such as organochlorines and neonicotinoids, plasticizers (phthalates and phenolic group that includes bisphenols, octylphenol, and nonylphenol), heavy metals like cadmium used in electrical batteries, and numerous molecules found in cosmetics such as benzophenones. The EDC activities of some of these commonly used molecules have now been detailed in the general population, and constitute a real societal problem. Therefore, industries have replaced these molecules by some equivalent, for instance Bis(2ethylhexyl) phthalate (DEHP) is replaced by di-isononyl phthalate (DiNP) or Bisphenol A (BPA) is replaced by Biphenol S (BPS) or Biphenol AF (BPAF). These replacement molecules are already found not only in water systems (for instance: BPAF 26.5 ng/L and BPS 6 ng/L), but also in aquatic organisms [11]. The main problem with these new molecules is that their biological effects have not yet been fully evaluated. Nevertheless, several results highlight the estrogenic activity of parent molecules nearly equivalent to BPA, using zebrafish as a model [12].

These EDCs are detected throughout the environment (water, sea, ground, sediments) and at concentrations ranging from ng to μg per liter in surface water. These compounds are not totally eliminated by waste water treatment plants and the diversity of the treatment processes suggests that the elimination of molecules will likely be different from one treatment station to another (activated sludge, ozonolysation, UV treatment, or detergent treatment) [13,14,15]. Because most potential endocrine disrupters have naturally low photolysis potency in water (Table 1), they could accumulate in the environment and therefore constitute a risk for humans and wildlife. Even when the molecules are degraded by photolysis and/or microorganisms, the metabolites could have endocrine-disrupting activities as well. Some natural hormones’ precursors can also be found in environment via their natural elimination [16]. The average excretion rates for some of these molecules such as androsterone (AD), dehydroepiandrosterone (DHEA), or α-diol are 3.3 mg/day, 1.2 mg/day, and 0.4 mg/day for men and 1.60, 0.6, and 0.2 mg/day for women, respectively [16]. Considering their ability to bind and activate directly or indirectly via their metabolites to steroid receptors, they can be considered endocrine-disrupting chemicals [17,18] and constitute a risk for humans and wildlife due to their concentration.

Several methods for the identification, detection, and quantification of molecules with endocrine activities and their metabolites have been developed up to now. They are mostly based on chromatography (gas and liquid) and mass spectrometry, associated with an efficient pre-concentration step (often Solid Phase Extraction) especially for water monitoring [19,20,21,22,23]. However, these methodologies do not give any information about the real biological activity of molecules. Therefore, the main challenge in the future will be the evaluation of the biological activity of water samples (Figure 1). Thus, it is essential to develop and use existing biological tools to evaluate the global endocrine activity of environmental water samples [24], mainly in order to determine an endocrine risk indicator [25].

## 3. Estrogen Receptors and Breast Cancer

In healthy breast tissue, a small proportion of the epithelial cells express ERs (10–15%). These so-called ER-positive cells respond to E2 by stimulating the proliferation of adjacent ER-negative cells through paracrine signaling, involving growth factors and cytokines. ERs are also responsible for the differentiation of the lobular structures, controlling the expression of genes related to the maintenance of the epithelial phenotype such as progesterone receptor, E-cadherin or the transcription factor MIST1. Two major subtypes of ERs (ERα and ERβ) have been identified in mammals. The proliferative effect of estrogens in breast tissue seems to be mediated only by ERα, as suggested by ERα knockout mice, in which the proliferative effect of estrogens on breast tissue is completely abolished.

**Figure 2:**
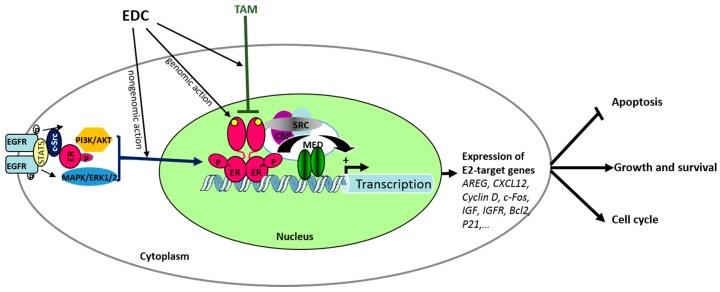
Ability of endocrine disrupter chemicals (EDCs) to alter estrogen signaling. At the cellular level, many EDCs can mimic the effects of estrogens through estrogen receptors (ERs). In the nucleus, EDCs are able to modulate transcription of E2-target genes (genomic action) by activating ER and the recruitment of cofactors such as CBP and SRC1, possessing intrinsic histone acetyltransferase activity, and the multiprotein mediators (MED) capable of modifying the chromatin organization of the specific gene promoter. In the cytoplasm, EDCs are able to interfere with pre-existing pathways (non-genomic action) altering interactions between ER and intracellular kinases (c-Src, MAPK, PI3K). EDCs like bisphenol A and diethylstilbestrol may induce rapid activation of MAPK or PI3 kinase signaling cascades, leading to ERK (extracellular-signal-regulated kinase) and AKT (Protein Kinase B) phosphorylations, intracellular calcium variations, or stimulation of cAMP production. Consequently, EDCs may influence cellular phenotypes, by genomic and non-genomic actions and responsiveness to antiestrogen tamoxifen (TAM) by changing the nuclear/cytoplasmic ER activity and expression, as well as the epigenetic and transcriptional regulations of various E2-target genes. EGFR: epidermal growth factor receptor.

The obvious side effects of the mitogenic action of E2/ERα on the breast epithelial cells is the probability of development and progression of breast cancer [45]. Indeed, about 70% of diagnosed breast tumors are ERα-positive and show estrogen-dependent growth and survival (Figure 2). Interestingly, ERβ can inhibit this growth effect by counteracting the ERα-dependent cell cycle progression. Genome-wide studies performed in cancer cell lines showed that growth stimulation by ERα involves activation of anti-apoptotic and pro-mitotic gene expression, while ERβ preferentially activates apoptotic signaling pathways [46,47]. It is also noteworthy that the ERα/ERβ ratio varies during the transition from the normal phenotype to the cancer phenotype. In fact, ERα expression increases in breast tumors, while the expression of ERβ decreases [48]. In addition to a direct effect on tumoral breast cells, it should be noted that E2 affects ERα-dependent tumor growth and metastasis via an increase in endothelial cells’ proliferation, neo-vascularization, and angiogenesis [49,50]. Regulation of the pro-angiogenic factor VEGF (vascular endothelial growth factor) and its receptor, VEGFR-1, as well as matrix metalloproteinases (MMPs) by estrogens, may represent one of the key molecular pathways responsible for the angiogenic effect of E2 during breast cancer development and progression [51,52].

ERs are ligand-activated transcription factors that interact with chromatin at specific sites to modulate the expression of hundreds of target genes (Figure 2). This ER–DNA interaction induces the mobilization of the transcriptional coregulators to modify chromatin compaction prior to the changes in gene expression [53]. However, ERs can also be activated in the absence of ligands by different mechanisms, notably by phosphorylation through activation of various growth factor receptors or protein kinases [54].

The expression of ERs in breast cancer is an important prognostic marker of endocrine therapy and overall survival. Indeed, ER-positive tumors are generally histologically more differentiated and show less metastatic potential than ER-negative cancers. Moreover, the presence of ERs provides the option of hormonal therapy based on anti-estrogenic treatments. These molecules are able to block the action of endogenous E2 by ER binding and to induce tumor growth arrest and regression. Some molecules such as fulvestrant (ICI 182,780, sold as Faslodex), are considered as pure antagonists which lead to the complete abolition of the estrogenic response. Other molecules called selective ER modulators (SERM), such as tamoxifen (Nolvadex) or raloxifen (Evista), have partial agonist/antagonist activities depending on the tissue, cell type, and target genes [55]. For over 30 years, tamoxifen has been the drug of choice for patients diagnosed with ER-positive breast tumors. It significantly reduces the risk of breast cancer recurrence and death. Also, unlike fulvestrant and aromatase inhibitors, tamoxifen can be used to treat breast cancer in both premenopausal and postmenopausal women and in men. Nevertheless, one-third of patients do not respond to the treatment or become resistant to tamoxifen treatment. This is generally associated with tumor aggressiveness and acquisition of an invasive phenotype. Various mechanisms have been proposed to explain antiestrogen resistance, such as loss of ER function or expression, or alterations in the expression of transcription co-regulators, microRNA, and growth factor signaling pathways that interact with ERs. In addition to the various endogenous factors affecting the development and cell phenotype of breast cancer, environmental EDCs are likely to modulate these responses as they may antagonize the antiestrogenic effects of tamoxifen by affecting the levels and activities of ERs or their transcriptional co-regulators (Figure 2). They can also act through epigenetic changes such as DNA methylation and histone modifications at a specific gene promoter. For example, tamoxifen resistance was often associated with hypermethylation of ER and PR genes, which is accompanied in a number of breast cancer cases by higher expression of DNA methyltransferase (DNMT). Thus, interferences with the ER signaling pathway during breast tumorigenesis may result in the acquisition of invasive and metastatic properties, and therefore result in poor clinical outcomes.

## 4. EDCs and Breast Cancer

Even though the role of EDCs in the increase of breast cancer incidence has not been clearly established, numerous studies point to the adverse effects of these compounds on this specific pathology. This is perhaps not surprising as EDCs share common structures with the natural hormone E2 (Table 2) and are able to enter the ER binding pocket [56].

### 4.1. Phenol Derivatives

Among the EDCs found in water, phenol derivatives such as BPA, BPS and other analogs, triclosan, alkylphenols, and benzophenones are the most prevalent. BPA has received the most attention. It is a chemical monomer used in plastics, canned food lining, and thermal receipt paper, to name a few applications. BPA is able to bind and activate ERα in most cell types investigated to date, including breast cancer cells. Consequently, BPA is considered an agonist of ERα and induces cell proliferation as well as E2-dependent gene expression [57]. Recently, the potential ER-transactivation ability of BPA as well as BPS and Bisphenol F (BPF) was confirmed using a zebrafish model [12,58]. In vivo, the reference dose for oral exposure (RfD) is set at 50 μg/kg/day, which is 1000 times lower than the lowest observable adverse effect level (LOAEL at 50 mg/kg/day) [59,60]. However, different effects on reproductive tissues have been observed in animals with doses lower than the RfD. For instance, prenatal exposure to BPA at 25 μg/kg/day increased the tumor susceptibility induced by 7,12-dimethylbenz[a]anthracene (DMBA) in mice, while adult exposure to the same low dose of BPA promoted ER-positive breast cancer growth in a xenograft model [59].

A study by Dhimolea et al. [61], using 250 μg/kg/day of BPA, showed modifications in DNA methylation throughout the genome of the offspring via in utero exposure. Some loci were hypomethylated while others were hypermethylated. The strongest difference between BPA-treated and vehicle-treated rats was observed at postnatal day (PND) 21, corresponding to the pre-pubertal stage in female rats and the beginning of production of E2 by the ovaries. In parallel to epigenetic changes, their transcriptomic analysis revealed major differences between BPA and control treatment at PND50, corresponding to the young adult stage [61]. A different study showed that in prepubescent rats exposed to 250 μg/kg/day of BPA (days 2–21 postnatally), the majority of genes were hypermethylated. Interestingly, 12 genes were homologous to human genes linked to the survival rate of patients with ER-positive breast cancer. Four genes were associated with a poor survival rate and eight were associated with a good survival rate. Among these genes, nine were hypermethylated in the distal region, indicating that their expression may be modified [62]. It should be noted that recent data suggest that BPA may act via ER-independent mechanisms including nuclear hormone receptors such as the thyroid hormone receptor, androgen receptor, and glucocorticoid receptor, but also with the orphan estrogen-related receptor gamma (ERRγ). BPA is indeed able to bind ERRγ with a high affinity in vitro [63], and the physiological importance of this interaction was demonstrated in vivo in a zebrafish model [64]. Surprisingly, there is still little information about the potential activation by endocrine disrupters of orphan receptors related to estrogen receptors; this topic should be investigated in more detail.

Another phenol derivative present in water is triclosan. It is a broad-spectrum antimicrobial agent used in several cosmetic products such as skin cream and toothpastes. Triclosan is approved by the European Commission for topical administration in humans at a concentration of 0.03% and its acceptable daily intake (ADI), 1/100 of the no-observed-adverse-effect-level (NOAEL), is estimated at 0.17 nmol/kg/day, a concentration often reached in drinking water. However, even if the amount of triclosan found in the environment does not reach the critical NOAEL value, higher concentrations of this compound can be found in human tissues, suggesting bioaccumulation and a potential impact on human physiology [36]. In vitro, triclosan induced proliferation of the ER-positive breast cancer cell line MCF-7 through the non-genomic ER signaling pathway, characterized by an increased phosphorylation of IRS-1, AKT, and MEK/ERK. Moreover, triclosan induced an increase of cyclin D1 and cyclin E and a decrease of p21 in MCF-7 cells, confirming the proliferating effect of this compound [65]. Triclosan induced cell migration and invasion with an increase of epithelial–mesenchymal transition (EMT) markers such as N-cadherin, similar to what was shown with E2 exposure. The phenotype induced by triclosan was inhibited by ICI 182,780, an ER inhibitor, suggesting an ER-dependent pathway [66]. In vivo, the effect of triclosan on tumoral MCF-7 cells proliferation in xenografted mice was confirmed by an increase in cyclin D1 and PCNA expression and a decrease in p21 staining. Moreover, triclosan repressed Bax expression and increased cathepsin D expression, in agreement with in vitro results showing an increase of metastatic phenotype and a decrease of apoptosis [65,67]. However, recent work on rats exposed to an environmental dose of triclosan (0.05 mg/kg/day) during a long period, from birth PND1 to reproductive stage and lactation (PND146), showed different results. Triclosan induced morphological changes of the mammary gland by increasing adipose tissue and decreasing the proportion of lobular tissue, but further transcriptomic and gene set enrichment analysis showed that genes upregulated by triclosan treatment during that exposure were homologous to genes downregulated in breast cancer in humans; conversely, genes downregulated by triclosan are homologous to certain genes upregulated in breast cancer [68]. These results suggest that the model species, timing of exposure, and/or dose can have significantly different effects; further studies should be undertaken to better understand how triclosan can affect the development of breast cancer.

Alkylphenols represent another group of phenol derivatives found in water. The two most documented are 4-octylphenol and 4-nonylphenol found in soil, air, and water, resulting from intense use in paints, plastics, or as a surfactant in agricultural products [67,69]. The human population is mostly exposed through diet. The concentration range of alkylphenol in the diet varies significantly between countries and types of foods. For instance, concentrations of nonyphenol found in mussels vary from a few nanograms to 180 μg/kg depending on the region of analysis. The concentration of 4-nonylphenol in oysters reached up to 236 μg/kg [70] in Taiwan, and was detected in the drinking water [71]. Estrogenic activity of 4-nonylphenol was determined over 20 years ago. It increases mitotic activity in MCF-7 cells and in the rat endometrium [72]. 4-nonylphenol induced cell proliferation and migration through the same mechanisms triggered by triclosan, including induction of cyclin D1 and cathepsin B and decrease of p21 expression. Similarly to triclosan, 4-nonylphenol induces tumor growth in MCF-7 xenograft model in nude mice [67,73]. Moreover, the expression profile of estrogen-responsive genes in breast cancer was established after treatment with E2 or different phenol derivatives. The results showed that the expression profile induced by 4-nonylphenol was very similar to E2 with a correlation coefficient *R* = 0.90, while the profile generated by octylphenol was less correlated (*R* = 0.75), albeit significant. The cluster analysis clearly showed an upregulation of genes linked to proliferation, transcription, and transport in E2 and 4-nonylphenol-treated cells [74]. A comparative study using different in vitro and in vivo tests in zebrafish showed that 4-nonylphenol was estrogenic in both approaches but with a half maximal effective concentration (EC50) much lower than E2, around 10,000 times [75]. In vivo, 25 mg/kg/day 4-nonylphenol provided orally enhances the E3 serum level via hepatic production, and this upregulation of estrogens might be involved in breast cancer susceptibility. However, a different chronic treatment experiment showed no effect of 4-nonylphenol on E3 serum level when provided at 30 mg/kg/day for 32 weeks. However, a parallel experiment with 45 mg/kg/day showed an increase of mammary cancer formation in MMTVneu mice that expressed an unactivated Erbb2 under the control of the mouse mammary tumor virus promoter that gave an increase in cancer susceptibility [69]. Concerning octylphenol, a study using 100 to 1000 ppm of octylphenol mixed into the diet during pregnancy showed an increase of early incidence and number of mammary cancers induced by DMBA [76]. Nevertheless, as for triclosan, the carcinogenic effects of octylphenol remain controversial.

Benzophenones (BP) are used as UV filters and found in watery environments. The major form is BP-3, which can be used up to 10% as an active ingredient in Europe and has been detected in surface water at concentrations up to 125 ng/L. BP-1 is the major metabolite of BP-3 and is found in the environment along with two other metabolites such as BP-8 and trihydroxybenzophenone (THB) [77]. Another benzophenone commonly detected is BP-4, reported at high concentrations in Switzerland [78]. BP-3 was described as a weak estrogenic compound in the MCF-7 cell line and induced proliferation in a dose–response relationship, with the highest activity at the highest dose at 50 μM [79]. Conversely, Nakagawa and Suzuki did not find any estrogenic effect of BP-3, even at 1 μM, the concentration where BP-1, BP-8, and THB showed the highest proliferation effect [73,80]. A screening of 10 benzophenones performed by our team showed a weak, but not statistically significant estrogenic induction of breast cancer cell proliferation by BP-3 and BP-1. However, our work showed a strong activity of 4-hydroxybenzophenone (4BP) and 4,4′-dihydroxybenzophenone (44′BP) and confirmed the effect of the two metabolites of BP-3, BP-8, and THB on cell proliferation and E2-dependent gene expression [81].

### 4.2. Neonicotinoids

Neonicotinoids belong to a class of neuroactive insecticides targeting the nicotinic receptor. Neonicotinoids have been reported in water samples since 2012, with a concentration range from 0.002 to 3.6 μg/L [82]. The use of this specific class of insecticide has come in for criticism because of the negative impact on the bee population. About 10 neonicotinoids are currently commercially available, but the most commonly used are imidacloprid, thiacloprid, and thiametoxan [83,84]. The effect of neonicotinoids, especially acetamiprid and imidacloprid, on neurons in invertebrates, but also in vertebrates, is relatively well characterized. For instance, it was shown that several neonicotinoids exerted an excitatory effect similar to nicotine via the activation of the nicotinic receptor, and more specifically the α-7-subunit of the nicotinic receptor, and could potentially impact human health [85]. However, the impact of neonicotinoids on breast cancer has not yet been fully assessed. Only one article referred to the effect of thiacloprid, imidacloprid, and thiametoxan in breast cancer [82]. More specifically, the biological effects of these three compounds were assessed on aromatase expression and activity. This enzyme metabolizes some androgens such as testosterone into estrogens and is a prime target in breast cancer therapy. The results showed that thiacloprid and thiametoxan, but not imidacloprid, induced aromatase expression and activity in a non-monotonic concentration–response relationship. It is noteworthy that this increase in aromatase expression involved two alternative promoters active in breast cancer cells but not in normal breast tissue [82]. Recently, it was shown that breast tumor cells are selectively stimulated via alpha-7 nicotinic receptor activation, increasing their migration capacity [86]. Thus, these results suggest that neonicotinoids could exert agonist effects on nicotinic receptors present on breast cancer cells and promote their migration, similar to what has been suggested for the nervous system. Further work should help us understand the potential effects of neonicotinoids on breast cancer cells.

### 4.3. Natural and Synthetic Estrogens

Natural estrogens E1, E2, and E3 are found in the environment. The two main sources of release is the human population, which discharges around 30,000 kg/year, and livestock, which discharges 83,000 kg/year [87,88]. E1 and E2 were found at high concentrations in Chinese drinking water treatment works, and can likely represent a risk to the human population [89]. Maybe the most important concern is the very high density of animals in Concentrated Animal Feeding Operations (CAFOs, as defined by the United States Department of Agriculture, USDA), which generate large amounts of manure, a significant source of hormone, into the soil and surface water. In addition to endogenous sex steroids found in manure as a natural physiological excretion process, over 90% of cattle in U.S. CAFOs receive steroid hormone treatment (implant or via feeding) for growth promotion [90]. The majority of the contaminated manure is used as fertilizer, without treatment, and surface runoff is likely to transport hormones from cropland to surface water [91,92]. Mansell and colleagues reported the presence of 17α-estradiol, 17β-estradiol, estrone, androstenedione, testosterone, and progesterone (ranging from 5 to 250 ng L^−1^) in runoff from feedlot surfaces during a rainfall simulation after the animals were removed from the pens [93]. Dairy effluent samples collected through the milking period in different New Zealand farms had total estrogen discharge ranging between 40 ng/L and 11700 ng/L [94]. Similarly, numerous studies have highlighted the presence of natural and synthetic estrogens (estrone, 17b estradiol, and estriol), androgens (trenbolone, and androgens receptor agonist up to 50 times more potent than testosterone), and progestagens (progesterone and melengestrol) in rivers close to CAFO, in the USA [95,96]. In 2000, the Joint FAO/WHO Expert Committee on Food Additives determined an ADI of 0–50 ng/kg of body weight, similar to NOAEL of 0.3 mg/day [97]. However, it should be noted that a recent study showed that higher urinary estrogen levels, including metabolites, were associated with a higher breast cancer risk in postmenopausal women, and the risk doubled between the lowest and highest percentile [98]. Surprisingly, even if overexposure to endogenous estrogens is suggested to be a significant risk factor, a definitive link between environmentally present natural estrogens and breast cancer incidence has not been demonstrated.

In contraceptive pills, the major synthetic hormones are EE2 and progestins, with the release of EE2 being estimated to reach 700 kg/year [87]. This environmental EE2 release may participate in the estrogen overexposure of the human population. At the same time, the environmental rates of progestins are not well documented. However, a study using several synthetic progestins on zebrafish in vivo and in vitro models showed that these compounds also have estrogenic activity and, consequently, affect the development of breast cancer [99].

In addition to anthropogenic and natural estrogens of vertebrate origin, emerging natural compounds found in water are phytoestrogens and mycoestrogens. The main compounds detected in surface water from different rivers are biochanin A (0–19 μg/L), genistein (0–2.65 μg/L), and daidzein (0–43 μg/L) for phytoestrogens and zearalenone (0–80 ng/μL) for mycoestrogens (for review, see [100]). Recently, we have characterized the biological effect of these compounds on breast cancer cell proliferation. Briefly, we showed that phytoestrogens induced cell proliferation at concentrations 100–1000-fold higher than E2 and that zearalenone was a powerful estrogenic compound with the same efficiency as E2 [101].

## 5. A Zebrafish Model for Predicting the Estrogenicity and Carcinogenesis of Chemicals in Aquatic Environments

Zebrafish are small and hardy fish with a relatively short generation time, and so are currently used in developmental biology, chemical screening, and toxicology studies. Zebrafish have recently become an effective model organism for studying carcinogenesis in ecotoxicological as well as medical research [53]. Human and zebrafish genome sequences show 70% common orthologous genes, including nuclear receptors, tumor suppressors, oncogenes, and cell-cycle genes as well as genes required for xenobiotic metabolism and biotransformation [102]. Thus, because of the similarity in biological pathways and molecular mechanisms between zebrafish and humans, zebrafish can be an alternative model to elucidate how deregulation of signaling pathways could contribute to pathophysiological functions. Lastly, zebrafish can be used as a predictive test in diagnostics of EDCs’ adverse effects in human health. For example, environmentally relevant doses of BPA induced endocrine disruption in the liver, brain, and reproductive organs in zebrafish [103]. These effects match those observed in mammals, as BPA interacts with the ERs in mammals and is capable of inducing the development and progression of several hormone-dependent cancers such as breast, ovary, and prostate [104,105,106].

**Figure 3:**
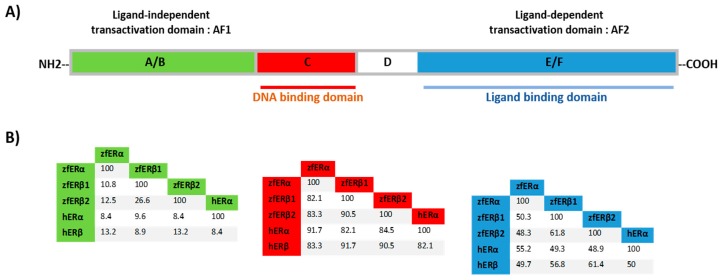
Schematic ER structure and the percentage identity between human ER (hER) and zebrafish ER (zfER) forms. (**A**) Different domains involved in DNA binding, ligand binding, and ligand-independent transactivation function (AF-1) and ligand-dependent transactivation function (AF-2) are indicated; (**B**) the percentage identity of coding regions of the N-terminal, DNA binding, and ligand binding domains between zfERα, zfERβ1, zfERβ2, hERα and hERβ, are indicated [107].

Previously, we cloned and characterized three distinct ER forms in zebrafish (zfERα, zfERβ1, and zfERβ2). As in mammals, zfERs are mainly expressed in E2-target tissues such as the ovaries, testes, liver, pituitary, and brain [107]. Sequence analysis demonstrated that, like in mammals, zfERs possess a hypervariable N-terminal domain. However, the serine residues corresponding to the phosphorylation sites, important for ER ligand-independent activation, are conserved. The DNA-binding domain of zfER forms shares above 90% of identity and possesses a P-box identical to that of human ERs, suggesting that they could bind to the same estrogen response element (ERE) (Figure 3). The ligand-binding domain of zfERs and human ERs shares a closely related structural organization and similar ligand selectivity and affinity (Figure 3, [107]). Moreover, Scatchard analysis showed that each zfER protein binds E2 with a high affinity similar to the values obtained in humans, with a dissociation constant (*Kd*) ranging between 0.42 nM to 0.75 nM. All three zfERs are able to activate E2-target genes in an E2-dependent manner with almost the same efficiency as human ERs, indicating that each form is able to specifically recognize ERE sequences [107].

Thus, the characterization of the zebrafish ER forms has provided a valuable tool to investigate ecotoxicological potential of thousands of chemicals produced by the industry and commonly found in the aquatic environment [108]. In a reconstituted glial cell-based assay (U-250 MG), we showed that the estrogenic potency of environmental chemicals differed markedly depending on the zfER subtype expressed in the assay. Moreover, we showed that the combination of environmental chemicals led to synergistic estrogenic potency, even when each single chemical was present at low concentrations, with reduced biological effect [109]. Cosnefroy et al. also reported the development of a new cell-based reporter gene assay established in a zebrafish hepatic cell line expressing zfERs (ZELH). This assay provides a fast and highly responsive test suitable for high-throughput screening of different chemical classes, including natural and synthetic estrogens, mycoestrogens, and industrial chemicals [110]. Notably, this study revealed that some benzophenone UV filters, detected in the environment, water, and fish tissue [111], are able to activate all three zfERs, as previously reported for human ERs expressed in different cancer cell lines [81,112].

Zebrafish embryos are also currently used to evaluate the impacts of in vivo exposure to aquatic chemicals on normal physiological processes. E2-target genes such as *ERs*, vitellogenin (*VTG*), and cytochrome P450 aromatase (*cyp19a1b*) are induced by xenoestrogens in embryonic–larval zebrafish and therefore are commonly used as endocrine biomarkers. Additionally, transgenic zebrafish models expressing an estrogen-dependent Green Fluorescent Protein (GFP)-based reporter gene were recently developed. These animals represent a powerful model to evaluate the in vivo and tissue-specific actions of EDCs present in water samples [113,114,115,116]. For example, using wild-type and transgenic Tg(cyp19a1b)-GFP embryo zebrafish, we showed that some progestins are able to exert additive effects to the environmental estrogens, therefore acting as estrogenic-like compounds. This resulted in the activation of the estrogen-specific marker, the *cyp19a1b* aromatase gene, in the developing zebrafish brain through the activation of ERs [99,117]. Furthermore, we have studied the effects of cadmium (Cd, a heavy metal) exposure on estrogen signaling in zebrafish. Cadmium is a naturally occurring element but also comes from tobacco smoke, industrial manufacturing (batteries), and agricultural activity. Cadmium is found in the atmosphere, food, soil, and water, and has been associated with multiple adverse health effects [118,119]. Cadmium is classified as a human carcinogen and long-term exposure is linked to increased risk of numerous cancers [120,121]. Our results in zebrafish demonstrated that Cd acts as a potent anti-estrogen in vivo and in vitro [122]. We showed that Cd inhibited the E2-induction of *cyp19a1b* aromatase gene, in an ER-dependent manner, in radial glial cells of zebrafish embryos. This inhibitory effect was accompanied by a significant downregulation of *zfER* gene expression in the developing brain. Interestingly, Cd-induced E2 antagonism can be reversed, at the protein level, by zinc supplementation [122].

Numerous laboratories have successfully used zebrafish models as a powerful in vivo assay to evaluate human cancer cell survival, migration, and metastasis [123,124,125,126,127,128]. Moreover, genetic manipulation methods such as sequence-specific nucleases, like TALEN and CRISPR/Cas9, have recently been used to generate gene knockout in transgenic zebrafish lines [129,130,131,132]. Furthermore, development of real-time in vivo optical imaging combining bioluminescence and fluorescence with high-resolution X-ray image acquisition allows us to characterize the in vivo dynamics of cellular processes and early changes in anatomical and molecular aspects of disease. Although these tests are usually performed in mice, zebrafish are an interesting model because their adaptive immune system is not developed until 14 days post-fertilization (dpf). In addition, in the context of screening studies for carcinogen chemicals or anti-cancer drugs, zebrafish represent a rapid and inexpensive model. For example, zebrafish models have recently been used to discover compounds that suppress melanoma development [133], or reduce the growth and migration of colorectal cancer [134]. Eguiara et al. demonstrated that BT-474 breast cancer cells treated with curcumin prior to injection into 2 dpf embryos showed significantly lower migration and mass formation compared to untreated cells [128]. Furthermore, from a high-throughput screening of 5000 molecules, Peterson et al. identified two compounds that completely suppressed the phenotypic effects of a zebrafish vascular mutation [135,136].

Altogether, this highlights the potential of zebrafish models for (i) discovering therapeutic compounds or toxicity of environmental chemicals that disrupt biological pathways and result in adverse effects; and (ii) identifying the mechanisms of action of these environmental chemicals.

It should be noted that zebrafish models can have different metabolism, pharmacodynamics, temperature, tissue anatomy, and behavior than mammals [137,138]. In addition, relatively few human mutations and their associated phenotypes have been studied in zebrafish to date. The whole genome of the teleost ancestor has also undergone a duplication, which is estimated to be more than 20% of all genes in the genome [139]. Thus, the relevance of zebrafish to human diseases should be relativized. Zebrafish should be considered an inexpensive and readily usable experimental model for molecule screening, but the results will need to be confirmed with mammalian studies.

## 6. Conclusions

Unraveling the effects and underlying mechanisms of endocrine disrupters on human health is a challenging task. However, some deleterious effects can be avoided by monitoring these environmental pollutants and by improving water quality, notably via the reduction of release or removal of these chemical compounds. To do this, we need to develop sensitive, efficient, and specific in vivo and in vitro bioassays to effectively characterize and quantify environmental pollutants, necessary steps to provide a legal limit to the presence of these man-made molecules. Several cell-based assays, developed previously [24], offer advantages in assessing the activity of chemicals in cancer cells and risks to normal cells. However, these current approaches are limited in their assessment of the effects of environmental chemicals. Lately, the zebrafish has become a powerful in vivo model for which new technologies (CRISPR/Cas9, in vivo imaging, etc.) are rapidly progressing in order to evaluate carcinogenic chemicals and anti-tumor drugs, as well as to study the effects of diet and diverse chemicals on tumor angiogenesis and metastasis. Furthermore, these new technologies will also contribute to a better understanding of the roles that certain genes and proteins can play in relaying the in vivo effects of chemical compounds.

## Figures and Tables

**Table 1:** Emerging endocrine-disrupting chemicals (EDCs) in water: description, half-life, and concentration.

Family	Molecule	Use	Half Life Photolysis in Distilled Water (Day)	Concentration in Surface Water (ng/L)	References
Neonicotinoids	Acetamiprid	Insecticide	34	20.6–23	[26,27]
	Clothiamidin		<1	100	[27,28]
	Nithiazine		<2	1000	[27,29]
	Imidacloprid		<1	20–10,400	[26,27,28,29]
	Nitenpyram		Na	Na	[26]
	Thiacloprid		10–63	Na	[26,27]
	Thiamethoxam		2.7–39.7	302	[27,28]
Phenolic compounds	Bisphenol A	Plasticizers	No degradation	0–56,000	[30,31,32]
	Octylphenol		0.6 to 2.5	<0.001–1440	[33,34]
	4-Nonylphenol (NP)		>2	0.006–32,800	[33,35]
	Triclosan (TCS)	Antibacterial/antifungal agent	37	Nd–3000	[36,37]
Benzophenones	BP1	Cosmetics	11.7	4.70	[38,39]
	BP3		38.9	10.3	[38,39]
	BP4		21.6	38.2	[38,39]
Natural hormones	E1	Natural hormones	55	Nd–180	[40,41]
	E2		60	Nd–175	[40,41]
	E3		40	Nd–94	[40,41]
Phthalate	Di(2-ethylhexyl) phthalate (DEHP)	Plasticizers	390–1600	Nd–197,000	[42,43]
	Dimethyl phthalate (DMP)		Na	Nd–31,000	[42]
	Diethyl phthalate (DEP)		Na	Nd–33,100	[42]
Heavy metal	Cadmium (Cd)	Batteries	Nd	9000–15,500	[44]
Drugs	EE2	Contraceptive pills	75	Nd–34	[40,41]
	Levonorgestrel		Nd	Nd–38	[19]

Na: not available, Nd: not determined.

**Table 2:** Structures of representative EDCs.

Family	Molecule	Structure
Neonicotinoids	Acetamiprid	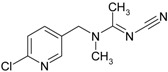
	Imidacloprid	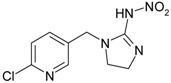
Phenolic compounds	Bisphenol A	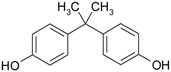
	Octylphenol	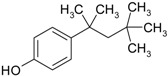
	Triclosan (TCS)	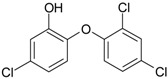
Benzophenones	BP3	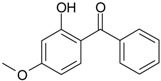
Natural hormones	E2	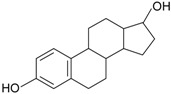
Phthalate	Di(2-ethylhexyl) phthalate (DEHP)	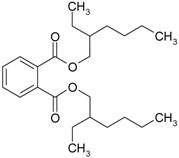
Drugs	Levonorgestrel